# SPY1 inhibits neuronal ferroptosis in amyotrophic lateral sclerosis by reducing lipid peroxidation through regulation of GCH1 and TFR1

**DOI:** 10.1038/s41418-022-01089-7

**Published:** 2022-11-28

**Authors:** Di Wang, Weiwei Liang, Di Huo, Hongyong Wang, Ying Wang, Chaohua Cong, Chunting Zhang, Shi Yan, Ming Gao, Xiaoli Su, Xingli Tan, Wenmo Zhang, Ling Han, Dongmei Zhang, Honglin Feng

**Affiliations:** 1 Department of Neurology, The First Affiliated Hospital of Harbin Medical University, Harbin, Heilongjiang Province PR China; 2 Department of Neurology, The Second Affiliated Hospital of Harbin Medical University, Harbin, Heilongjiang Province PR China; 3 Department of Neurology, Shanghai JiaoTong University School of Medicine, Shanghai No. 9 People’s Hospital, Shanghai, PR China; 4 Department of Neurology, The First Affiliated Hospital of USTC, Division of Life Science and Medicine, University of Science and Technology of China, Hefei City, Anhui Province PR China; 5 Harbin Veterinary Research Institute, Chinese Academy of Agricultural Sciences, Harbin, Heilongjiang Province PR China

**Keywords:** Neuroscience, Gene regulation

## Abstract

Ferroptosis is an iron-dependent cell death with the accumulation of lipid peroxidation and dysfunction of antioxidant systems. As the critical regulator, glutathione peroxidase 4 (GPX4) has been demonstrated to be down-regulated in amyotrophic lateral sclerosis (ALS). However, the mechanism of ferroptosis in ALS remains unclear. In this research, bioinformatics analysis revealed a high correlation between ALS, ferroptosis, and Speedy/RINGO cell cycle regulator family member A (SPY1). Lipid peroxidation of ferroptosis in hSOD1G93A cells and mice was generated by TFR1-imported excess free iron, decreased GSH, mitochondrial membrane dysfunction, upregulated ALOX15, and inactivation of GCH1, GPX4. SPY1 is a “cyclin-like” protein that has been proved to enhance the viability of hSOD1^G93A^ cells by inhibiting DNA damage. In our study, the decreased expression of SPY1 in ALS was resulted from unprecedented ubiquitination degradation mediated by MDM2 (a nuclear-localized E3 ubiquitin ligase). Further, SPY1 was identified as a novel ferroptosis suppressor via alleviating lipid peroxidation produced by dysregulated GCH1/BH4 axis (a resistance axis of ferroptosis) and transferrin receptor protein 1 (TFR1)-induced iron. Additionally, neuron-specific overexpression of SPY1 significantly delayed the occurrence and prolonged the survival in ALS transgenic mice through the above two pathways. These results suggest that SPY1 is a novel target for both ferroptosis and ALS.

## Introduction

Amyotrophic lateral sclerosis (ALS) is a fatal neurodegenerative disease that involves upper and lower motor neurons (MNs). The manifestation characterized by progressive muscle weakness and muscle atrophy frequently ends in respiratory failure. Until now, edaravone and riluzole are the only drugs approved by FDA but with limited effect. Mutations in genes such as superoxide dismutase 1 (SOD1), C9ORF72, TDP43, FUS/TLS, OPTN, TBK1, GRN, NEK1, and C21ORF2 contribute to the etiology [1]. The pathogenesis has been revealed to be related to oxidative stress, mitochondrial dysfunction, hyperexcitability, impaired protein homeostasis, axonopathy, aberrant RNA metabolism, dysregulated vesicle transport, impaired DNA repair, nuclear export, and glial dysfunction [2]. Mutant hSOD1^G93A^, the earliest and most widely used model, presents the complex pathology of ALS [3]. Although lots of mechanisms have been studied, there is still a lack of effective treatment implying that current mechanisms can not well interpret the reasons for occurrence and development of ALS.

Unlike other programmed death, ferroptosis is an iron-dependent cell death with accumulation of lipid peroxidation and dysfunction of antioxidant systems [4]. The main recognized endogenous mechanisms against lipid peroxidation consist of glutathione peroxidase 4 (GPX4), ferroptosis suppressor protein 1 (FSP1), and tetrahydrobiopterin (BH4) restrictively synthesized by GTP cyclohydrolase-1 (GCH1) [5]. Interestingly, iron metabolism disorder is a common feature of almost all neurodegenerative diseases confirmed by blood samples, imaging, and histopathology [6]. As a strong oxidant, abnormally accumulated iron will generate highly reactive hydroxyl radicals, especially through the Fenton reaction [7]. However, the administration of antioxidants alone cannot delay the development of ALS [8]. In recent years, various iron chelators have been shown to prolong the survival in SOD1-mutant transgenic mice [9, 10]. Deferoxamine (DFO), a ferroptosis inhibitor, once was used as an iron chelator to be tested in neurodegenerative diseases such as Huntington’s disease (HD) and Parkinson’s disease (PD) [11, 12]. Moreover, deferiprone has already entered the phase II clinical trial of ALS (NCT03293069). On the contrary, edaravone has displayed restraint for ferroptosis through reducing lipid peroxidation and the same as CuII(ATSM), which is effective in the initial clinical trials of ALS [13, 14]. As an essential regulator of ferroptosis, deletion of GPX4 has been found to induce degeneration of MNs and summarize the main characteristics of ALS [15]. Furthermore, the down-regulated expression of GPX4 in ALS mice and patients has been validated, and the course can be delayed by overexpression of GPX4 [16–18]. Nevertheless, systematic research on the mechanism of ferroptosis in ALS remains blank compared with other neurodegenerative diseases. And whether there are other mechanisms independent of GPX4 involved is still unknown.

Speedy/RINGO cell cycle regulator family member A (SPY1) is a highly conserved “cyclin-like” protein, previously considered to be associated with several cancers [19–22]. It contributes to CDK2 activation without promoting CDK2 phosphorylation, by inducing a conformation change of the CDK2 T-loop that obstructs the substrate-binding cleft prior to kinase activation [23]. Functions majorly include cell proliferation, chromosome depolymerization, apoptosis, cell cycle checkpoint activation, and DNA damage response (DDR). Intersections between DDR and ferroptosis have been found in different tumors. The silencing of DDR crucial molecules such as ATM provides a robust protective phenotype for ferroptosis [24]. P53 has been continuously demonstrated to affect ferroptosis, and its classical upstream E3 ubiquitin-protein ligase MDM2 also regulates ferroptosis in a p53-independent manner [25, 26]. In the previous study, we first showed that the expression of SPY1 has a significant decrease in ALS. Furthermore, overexpression of SPY1 can increase the viability of hSOD1^G93A^ cells by inhibiting the activation of ATM, ATR, and P53 in DDR [27]. However, an expression correlation between SPY1 and the ferroptosis suppressor GCH1 was found in the preliminary bioinformatics analysis of this study. It is still unclear whether there is a regulatory relationship between SPY1 and ferroptosis, and whether they will affect the progression of ALS.

In this research, we identified multiple mechanisms of ferroptosis in pathogenesis of ALS and clarified MDM2 accounting for ubiquitination degradation of SPY1. Furthermore, we confirmed that SPY1 was a novel regulator to inhibit ferroptosis by regulating GCH1/BH4 and transferrin receptor protein 1 (TFR1). These results provide new ideas for exploring the pathogenesis and treatment in ALS.

## Materials and methods

### Acquisition of dataset and data processing

The expression profiling by array was derived from series GSE7493 of the Gene Expression Omnibus (GEO, http://www.ncbi.nlm.nih.gov/geo/) database. Total RNA was extracted from embryonic MNs isolated from the ventral spinal cord of e14 mutant SOD1^G93A^ overexpressing rat embryos compared to wildtype SOD1 overexpressing embryos, then followed by a p75-antibody purification in combination with magnetic beads [28]. The probe ID was annotated by GPL1355 (Affymetrix Rat Genome 230 2.0 Array).

The transcriptome data contained 4 mutations (MU) and 5 wildtypes (WT) and was divided into 4 highs (High) and 4 lows (Low) according to the expression of SPY1. The original expression matrix was normalized with log2 and processed by R.

The ferroptosis dataset was downloaded from FerrDb (http://www.datjar.com:40013/bt2104/) which collects published drivers, suppressors, markers, inducers, inhibitors, and diseases associated with ferroptosis [29].

### Analysis for functional enrichment and differential expression

The normalized expression matrix was analyzed by Gene set enrichment analysis (GSEA) Linux 4.1.0 software using the ferroptosis dataset to obtain the gene list, and then processed with the GSEA package in RStudio.

The limma package was used to identify the differential expressed genes (DEGs) in MU vs. WT and SPY1 High vs. Low. The cut-off criteria for statistical significance were |logFC| ≥ 1 and *p* < 0.05. Since there were four probes representing SPY1, in two of which logFC > 0 while logFC < 0 in the other. To avoid omission, we took the average value of normalized data in these two groups respectively followed by differential expression analysis. The volcano plot using the ggplot2 package with geom point function displayed DEGs in MU vs. WT, while the heatmap using complex heatmap package was for SPY1 High vs. Low.

DAVID v6.8 (http://david.ncifcrf.gov) provided DEGs in SPY1 High vs. Low with the gene ontology (GO) analysis, including annotation for biological process (BP), molecular function (MF), and cellular component (CC) [30].

Pearson analysis was tested to confirm the correlation of DEGs.

### Reagents and antibodies

The reagents diluted according to the manufacturer’s instructions were as follows: z-VAD-fmk (Zvad, Promega, G7231), Necrostatin-1 (Nec-1, Sigma-Aldrich, N9037), Deferoxamine (DFO, Sigma-Aldrich, D9533), Ferrostatin-1 (Fer-1, Sigma-Aldrich, SML0583), RSL3 (Selleck, S8155), Erastin (Selleck, S7242), MG-132 (Enzo Life Science, BML-P102), pepstatin A (Sigma-Aldrich, P5318), E-64d (Sigma-Aldrich, E8640), (6 R)-5,6,7,8-tetrahydro-L-Biopterin (BH4, Cayman, 81880).

The antibodies diluted according to manufacturer’s were as follows: rabbit anti-ALOX15 (Abcam, ab244205), rabbit anti-FSP1 (Invitrogen, PA5-103183), rabbit anti-GDF15 (Abcam, ab105738), rabbit anti-GCH1 (Invitrogen, PA5-103865), rabbit anti-GPX4 (Abcam, ab125066), rabbit anti-SPY1 (Invitrogen, PA1-16959), rabbit anti-MDM2 (Proteintech, 17914-1-AP), rabbit anti-NEDD4 (Abcam, ab240753), rabbit anti-SKP2 (Abcam, ab183039), rabbit anti-TFR1 (Abcam, ab84036), rabbit anti-P53 (Proteintech, 10442-1-AP), rabbit anti-SP1 (Abcam, ab227383), rabbit anti-4-HNE (Abcam, ab46545), mouse anti-SMI32 (Santa Cruz, sc-133165), rabbit anti-Ubiquitin (Proteintech, 10201-2-AP), mouse anti-FLAG (Sigma-Aldrich, F1804), mouse anti-HA (Sigma-Aldrich, H3663), mouse anti-His (Abcam, ab18184), mouse anti-β-actin (Sigma-Aldrich, A5316), IRDye 800CW goat anti-rabbit IgG (Li-COR, 926-32210), IRDye 800CW goat anti-mouse IgG (Li-COR, 926-32211), HRP-labeled goat anti-rabbit IgG (Beyotime, A0208), HRP-labeled goat anti-mouse IgG (Beyotime, A0216), Alexa Fluor 488 labeled Goat anti-mouse IgG (ZSGB-BIO, ZF-0512), Alexa Fluor 594 labeled Goat anti-rabbit IgG (ZSGB-BIO, ZF-0516).

### Cell culture and model establishment of ALS

NSC34, the motor neuronal cell line (Cedarlane Laboratories, Vancouver, Canada), is a hybrid of embryonic mouse spinal cord motor neuron and neuroblastoma cell line. It is characterized by extension of neuritis, generation of action potentials, expression of neurofilament proteins and choline acetyl-transferase, synthesis and storage of acetylcholine, and induction of twitching in co-cultured muscle cells [31]. The ALS cell model was established by stably transfection of mutant hSOD1^G93A^ in NSC34, while the control was transfected with wild-type hSOD1 (WT) or empty puromycin lentivirus vector (EV).

SHSY5Y (human neuroblastoma), Hela (human cervical cancer), and HEK293T (human embryonic kidney) cells were obtained from the Cell Bank of Chinese Academy of Sciences.

The cells have been authenticated by short tandem repeat (STR) profiling and tested for mycoplasma contamination. The cells were cultivated in Dulbecco’s modified Eagle’s medium (DMEM, Gibco) supplemented by 10% fetal bovine serum (FBS, Natocor) and 100 U/mL of penicillin-streptomycin at 37 °C with 5% CO2.

### Measurement of cell viability and cytotoxicity

The cell viability was evaluated by the cell counting kit-8 (CCK8, Beyotime, C0038) according to the manufacturer’s instructions. The absorbance was assessed at 450 nm by a microplate reader (BioTek Instruments, Winooski, VT, USA). Similarly, LDH cytotoxicity assay kit (Beyotime, C0017) was used to detect cell cytotoxicity in cells, and the absorbance was assessed at 490 nm by a microplate reader with multiscan spectrum (Enspire, PE, USA).

### Measurement of lipid peroxidation, labile iron pool, and GSH

Cells were orderly stained with C11-BODIPY 581/591 fluorescence probe (1 μM, Invitrogen, C10445) and Hoechst 33342 (Invitrogen, H1399) for 30 min. The extent of lipid peroxidation was represented as the ratio of GFP(484/510 nm) to RFP (581/610 nm) fluorescence detected by the inverted fluorescence microscope (EVOS FL, AMG, USA) and quantified by ImageJ. Fluorescence was also measured by channel 488 Grn using the multiparameter flow cytometer (A60-Universal, Apogee, Britain), and the data were analyzed by FlowJo v10.8.1.

Accompanied by DFO chelating iron, Calcein-AM (1 μM, Invitrogen, C3099) was used to assess the labile iron pool (LIP). After incubation of Calcein-AM for 15 min, the fluorescence intensity at 496 nm (excitation wavelength) and 520 nm (emission wavelength) of initial and after iron chelation were measured by the microplate reader (Enspire, PE, USA) were recorded, respectively. The discrepancy between the latter and the former was indicated as the relative content of LIP.

The ratio of GSH/GSSG was tested by GSH and GSSG Assay Kit (Beyotime, S0053) according to the manufacturer’s instructions.

### Morphology of cell and mitochondria

The cell morphology was observed using an inverted fluorescence microscope (EVOS FL, AMG, USA). Transmission electron microscopy (HITACHI-H7650) was used to examine the morphological changes of mitochondria in cells and spinal cord tissues.

### Immunoblotting and immunoprecipitation

The cleaned cells and tissues were lysed by RIPA (Beyotime, P0013B) with the addition of PMSF (Beyotime, ST506) for 1 h at 4 °C. For immunoprecipitation, protein A + G magnetic beads (Beyotime, P2108) were incubated to remove nonspecific binding for 4 h at 4 °C. After incubation with antibodies overnight at 4 °C, the immunoprecipitates were separated by binding with beads for another 2 h at 4 °C and adequate washing. After concentration determination by the BCA protein assay kit (Beyotime, P0012), equal amounts of protein were transferred to the nitrocellulose membrane by 4–20% SDS-PAGE and transmembrane. Having finished blocking, the membrane was incubated with specific primary antibodies overnight at 4 °C. Subsequently, the rewarmed membrane was incubated with corresponding IRDye800-conjugated secondary antibodies for 1 h at room temperature. The visualization of blots was detected by the Odyssey infrared imaging system (Li-COR Biotechnology, Lincoln, NE, USA) and quantified by ImageJ.

### In vivo and in vitro ubiquitination assay

For in vivo ubiquitination assay, cells were co-transfected with SPY1-Flag, MDM2-HA, and Ub-His for 24 h. Subsequently, cells were incubated with MG132 (10 μM) for 4 h to block proteasome degradation. For in vitro ubiquitination assay, SPY1-Flag (1 μg), MDM2-HA (1 μg), and Ub-His (2 μg) proteins which were expressed and purified in *E. coli* Rosetta (DE3) were incubated with E1 (50 nM) and E2 (500 nM) in a 30 μl ubiquitination assay buffer (20 mM HEPES, pH 7.2, 5 mM MgCl_2_, 0.1 mM DTT, and 1 mM ATP) at 30 °C for 2 h. Subsequent immunoprecipitation and immunoblotting experiments were performed as described previously using anti-ubiquitin and anti-His antibodies.

### Plasmids, small-interfering RNA, and transfection

The SPY1 overexpression plasmid, empty vector with Flag-tag (SPY1-Flag and Flag), the SKP2, NEDD4, MDM2, GCH1, TFR1, P53 overexpression plasmids, empty vector with HA-tag (SKP2-HA, NEDD4-HA, MDM2-HA, GCH1-HA, TFR1-HA, P53-HA, and HA), and the Ubiquitin with His-tag (Ub-His) were purchased from Genscript (Nanjing, China). The designed plasmids expressing different domains of SPY1, wildtype of SP1, and SP1(S59A) were synthesized by Genscript (Nanjing, China). The small-interfering RNA targeting MDM2, SPY1, GCH1 (SiMDM2, SiSPY1, and SiGCH1), and control (SiNC) were synthesized by GenePharma (Shanghai, China). The siRNA sequences used were as follows:

SiMDM2, 5’-GCC AUU GCU UUU GAA GUU AUU-3’ and 5’-UAA CUU CAA AAG CAA UGG CUU-3’.

SiSPY1, 5’-CCA GAG GGC CUU GUC UAA UTT-3’ and 5’-AUU AGA CAA GGC CCU CUG GTT-3’.

SiGCH1, 5’-UAC AAG UAC CUA ACU UUA CUU-3’ and 5’-GUA AAG UUA GGU ACU UGU AAU-3’.

SiNC, 5’-UUC UCC GAA CGU GUC ACG UTT-3’ and 5’-ACG UGA CAC GUU CGG AGA ATT-3’.

The plasmids and siRNA (3 μg) were used to transiently transfect cells assisted by Lipofectamine2000 (Invitrogen, 11668030). Briefly, incubated with transfection for 4-6 h, cells were maintained in low-serum culture for 48 h to accomplish interfering expression for subsequent experiments.

### Detection of mRNA level

The mRNA levels of target genes were detected by quantitative real-time polymerase chain reaction (qRT-PCR) as previously described [32]. The mouse primer sequences used in this study were as follows:

ALOX15, 5′-GACACTTGGTGGCTGAGGTCTT-3′ (forward) and 5′-TCTCTGAGATCAGGTCGCTCCT-3′ (reverse).

FSP1, 5′-GCGACCTTCAAGGACAACTTCC-3′ (forward) and 5′-GCCAGGATAAGATGTGAGAAGGG-3′ (reverse).

GDF15, 5′-AGCCGAGAGGACTCGAACTCAG-3′ (forward) and 5′-GGTTGACGCGGAGTAGCAGCT-3′ (reverse).

GCH1, 5′-AGCAAGTCCTTGGTCTCAGTAAAC-3′ (forward) and 5′-ACCGCAATCTGTTTGGTGAGGC-3′ (reverse).

GPX4, 5′-CCTCTGCTGCAAGAGCCTCCC-3′ (forward) and 5′-CTTATCCAGGCAGACCATGTGC-3′ (reverse).

SPY1, 5′-AAGGGACCAACTCTGGGACAGA-3′ (forward) and 5′-ACAGCTCCACTGTGATGCACAG-3′ (reverse).

TFR1, 5′-GAAGTCCAGTGTGGGAACAGGT-3′ (forward) and 5′-CAACCACTCAGTGGCACCAACA-3′ (reverse).

DMT1, 5′-TTGCAGCGAGACTTGGAGTGGT-3′ (forward) and 5′-GCTGAGCCAATGACTTCCTGCA-3′ (reverse).

FPN1, 5′-TTGGCAGGTGCTAGAAGGAAG-3′ (forward) and 5′-CAGGCCGGACATGACTTGTA-3′ (reverse).

β-actin, 5′-CCAGCCTTCCTTCTTGGGTAT-3′ (forward) and 5′-TGCTGGAAGGTGGACAGTGAG-3′ (reverse).

### Prediction of E3 ligase for SPY1 ubiquitination

UbiBrowser (http://ubibrowser.ncpsb.org) is an integrated bioinformatics platform to predict and present the proteome-wide human E3-substrate interaction network based on multiple types of heterogeneous biological evidence including homology E3-substrate interaction, enriched domain and GO term pair, protein interaction network loop, and inferred E3 recognition consensus motif [33]. On account of the high homology and conservation, SPY1 was regarded as a substrate to predict the interacted E3 ligase using the UbiBrowser.

### Measurement of mitochondrial lipid peroxidation and membrane potential

Cells were treated with 3-[4-(Perylenylphenylphosphino) phenoxy] propyltriphenylphosphoniumio-dide (MitoPeDPP, 0.1 μM, Dojindo, M466) for 30 min at 37 °C. The fluorescence of mitochondrial lipid peroxidation was measured by channel 488 Grn using the multiparameter flow cytometer (A60-Universal, Apogee, Britain), And analyzed by FlowJo v10.8.1.

Mitochondrial membrane potential (MMP) was detected through incubation with the JC-1 fluorescence probe (5 μM, Abcam, ab141387) but followed by the analysis using a flow cytometer (FC500, Beckmancoulter, USA). JC-1 aggregates showed double-positive for FL1 (488/525 nm) and FL2 (488/585 nm) suggesting the normal MMP, and monomers showed single-positive for FL1 implying the decrease of MMP. The data were analyzed by FlowJo v10.8.1.

### Measurement of BH4

BH4 and oxidized biopterins (BH2) were evaluated by high-performance liquid chromatography (HPLC) followed by electrochemical and fluorescent detection respectively as described previously [34].

### Experimental animals and behavioral evaluation

Mutant hSOD1^G93A^ overexpressing (B6SJL-Tg-SOD1^G93A^-1Gur/J) transgenic mice purchased from Jackson Laboratory (Stock no. 002726; Bar Harbor, ME, USA) were bred with non-transgenic C57/BL6 mice to continue species. The genotypes were determined by PCR which is blind in the study. The littermates not expressing hSOD1^G93A^were regarded as wild-type controls (WT). All experimental animals were conducted according to the protocol approved by the Harbin Medical University Experimental Animal Research Ethics Committee. The size of each group (*n* = 10, randomly assigned) is based on the preclinical study guidelines recommended by the ALS Institute of Therapeutic Development.

The model of hSOD1^G93A^ mice with overexpressed SPY1 was established by intracerebroventricular injection of recombinant viruses targeting neuron-specific expression. In brief, the adeno-associated virus 9 (AAV9) carrying neuronal-specific promoter and overexpressed SPY1 (AAV9-hSyn-SPY1-mCherry) and control (AAV9-hSyn-mCherry) purchased from Genechem (Shanghai, China) were injected (10^8^ TU/ml, 5 μl) into the lateral ventricle of hSOD1^G93A^ mice at 60 d. The injection point is 1.5 mm beside the anterior fontanelle, 1.1 mm in the direction of posterior fontanelle and 2.0 mm deep. Then their expression was verified by fluorescence of cherry at 90 d.

The evaluation of mice behavior consisted of weight changes, rotarod retention, onset time, and survival period as Guidelines for the preclinical in vivo evaluation of pharmacological active drugs for ALS/MND described. Body weight and rotarod retention were measured every other week. The onset was determined by the occurrence of rotating retention for less than 180 s. And the endpoint of mice defined as being unable to stand upright within 30 s after lying on one side was recorded.

### Immunohistochemistry and immunofluorescence

Tissues of gastrocnemius muscle and lumbar spinal cord isolated from mice at 150 d were used for immunohistochemistry and immunofluorescence as described previously [35]. Briefly, the frozen section (8 μm) of gastrocnemius was stained with hematoxylin and eosin (H&E). The paraffin section (5 μm) of the spinal cord was stained orderly with specific primary and HRP-labeled secondary antibodies, then visualized by the DAB horseradish peroxidase color development kit (Beyotime, P0202) for immunohistochemistry. The images were obtained using the microscope (Leica, Wetzlar, Germany) and quantified by Image-Pro Plus. For immunofluorescence, sections were incubated with fluorescence-labeled secondary antibodies followed by the antifade mounting medium with DAPI (Beyotime, P0131). The images were captured using the inverted fluorescence microscope (Leica, Wetzlar, Germany).

### Statistical analysis

All the data were tested for normal distribution. Based on at least three independent experiments, data were presented as mean ± standard deviation (SD). Variance between groups was similar as assessed by Bartlett’s test for one-way ANOVA. The differences between two groups were determined by the student’s t-test. The differences between multiple groups were analyzed by one-way or two-way ANOVA followed by Tukey’s post hoc tests. *p* < 0.05 was considered statistically significant. The analysis of Pearson correlation and Kaplan-Meier survival were performed using the software of GraphPad Prism 8 (San Diego, CA, USA).

## Results

### Transcriptome data of primary rat neurons reveals an association between ALS, ferroptosis, and SPY1

**Fig. 1:**
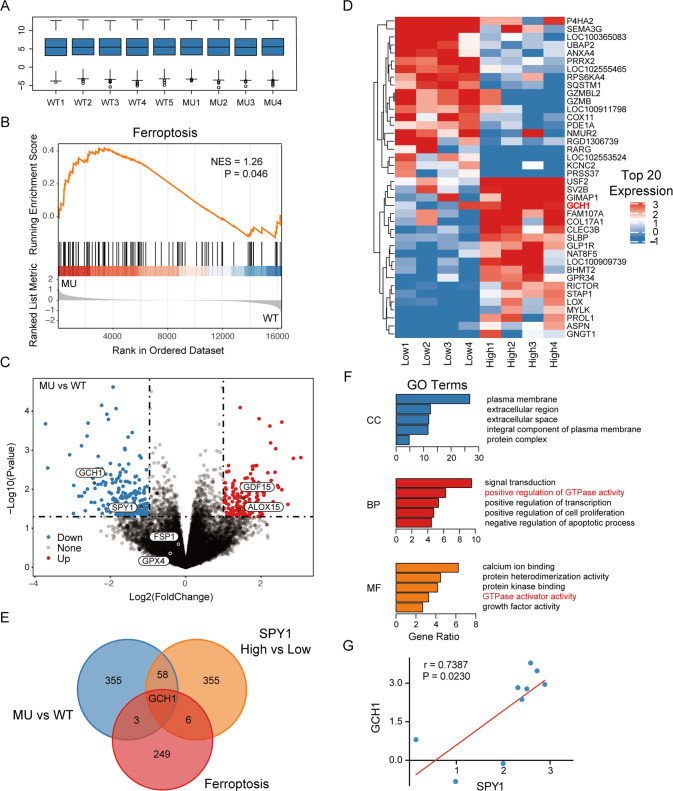
The association between ALS, ferroptosis, and SPY1 is uncovered in the transcriptome data of primary rat neurons. The transcriptome data of GSE7493 abstracted from embryonic MNs in the SOD1^G93A^ mutant model of ALS was downloaded for analysis. **A** Standardization with log2 for the data, including 4 wildtypes (WT) and 5 mutants (MU). **B** GSEA for ferroptosis in MU vs WT (NES = 1.26, *p* = 0.046). **C** Differential expression analysis in MU vs WT containing 193 up-regulated genes and 224 down-regulated genes (*p* < 0.05, |logFC | ≥ 1). **D** The data were divided into groups of 4 lows and 4 highs based on the expression of SPY1. The heatmap exhibited the top 20 DEGs in SPY1 High vs. Low (*p* < 0.05, |logFC | ≥ 1). **E** The Venn diagram overlapped 417 DEGs in MU vs. WT, 420 DEGs in SPY1 High vs. Low, and 259 ferroptosis-related genes. **F** DEGs in SPY1 High vs Low were performed enrichment in the GO terms of CC, BP, and MF via David. The bar chart showed the top 5 of each term sorted by gene ratio (*p* < 0.05). **G** Pearson correlation analysis was performed on the expression of SPY1 and GCH1 (*r* = 0.7387, *p* = 0.0230). Statistical analysis by Student’s *t*-test.

For the preliminary screening of related mechanism and target genes in ALS, GSE7493 (including 4 MU and 5 WT) from the GEO database was selected for analysis. Firstly, the transcriptome data from embryonic motor neurons of SOD1^G93A^ rats was performed standardization to reduce the impact of individual deviations (Fig. 1A). Next, all 16324 genes were enriched by the GSEA tool using the ferroptosis-related gene set downloaded from FerrDb. The ALS transcriptome data showed significant enrichment in ferroptosis (NES = 1.26, *p* = 0.046) (Fig. 1B). To identify the target genes, gene differential expression analysis was performed. A total of 417 differentially expressed genes (DEGs) were screened. Among them, SPY1 (LogFC = −1.175, *p* = 0.028) was confirmed to be down-regulated (Fig. 1C) as previously detected in mice and cells [27]. Subsequently, to identify potential effector genes of SPY1 involved in ALS, the transcriptome data were divided into SPY1 high and low expression groups. 215 up-regulated genes and 205 down-regulated genes (*p* < 0.05, |LogFC| ≥ 1) were screened (Fig. 1D). Through further intersection with DEGs in MU vs. WT, SPY1 High vs. Low, and ferroptosis-related gene set, the GTP cyclohydrolase GCH1 was the only gene to link to ALS, ferroptosis, and SPY1 (Fig. 1E). Moreover, the DEGs in SPY1 High vs. Low were functionally annotated for biological process, cell composition, and molecular function by David, indicating that GTPase activity was also associated with SPY1 expression (Fig. 1F). Finally, the direct positive correlation between SPY1 and GCH1 was identified by Pearson correlation analysis (*r* = 0.7387, *p* = 0.0230) (Fig. 1G). These bioinformatic results suggest that SPY1 may play a role in affecting ferroptosis in ALS through GCH1.

### Ferroptosis is involved in the cell model of ALS

**Fig. 2:**
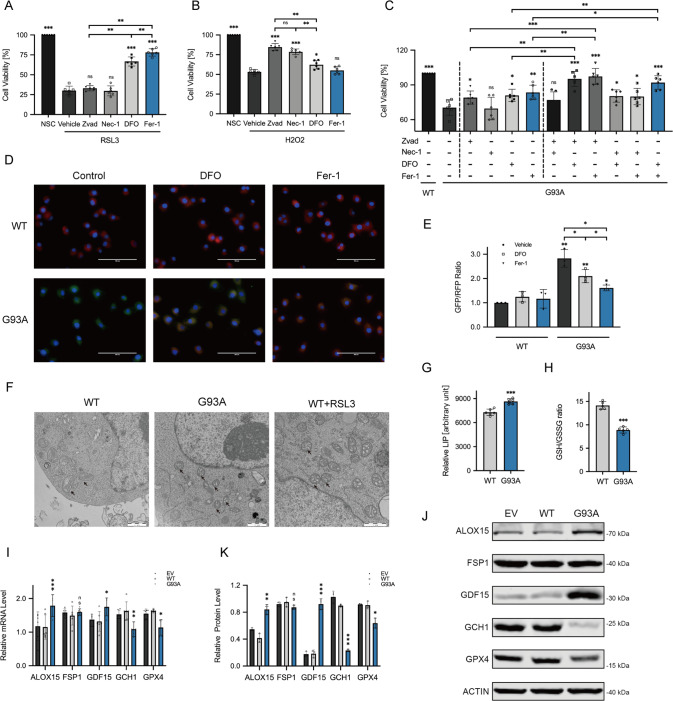
Evidence of ferroptosis is found in the ALS cell model. Cells were incubated with Zvad (20 μM), Nec-1 (50 μM), DFO (100 μM), Fer-1 (10 μM), and vehicle for 24 h. The cell viability was assessed by CCK8 assay. **A** The bar chart showed different inhibitors’ effects on the viability of NSC34 cells with induction of 2 μM RSL3 for 2 h (*n* = 6). **B** Different inhibitors’ effects on the oxidative stress model mediated by administration of H_2_O_2_ (200 μM) for 24 h in NSC34 cells (*n* = 6). **C** The effect of each alone or any two of four inhibitors combined on hSOD1^G93A^ and WT cells (*n* = 6). **D**, **E** C11-BODIPY immunofluorescence staining was displayed in representative microscopy images. Scale bar, 100 μM. Fluorescence which shifts from red to green in response to oxidation, was quantified by the ratio of GFP (484/510 nm) to RFP (581/610 nm) using ImageJ, indicating the extent of lipid peroxidation (*n* = 3). **F** Electron microscopy images exhibited morphological changes in mitochondria. Scale bar, 1 μM. **G** The relative content of LIP was assessed to compare with hSOD1^G93A^ and WT cells using Calcein-AM and DFO assay (*n* = 6). **H** The GSH and GSSG Assay Kit were used for the detection of GSH in hSOD1^G93A^ and WT cells. The relative content was represented by the ratio of GSH/GSSG (*n* = 6). **I** The relative mRNA level of ALOX15, FSP1, GDF15, GCH1, and GPX4 in hSOD1^G93A^ cells compared with control was measured by qRT-PCR (*n* = 6). **J**, **K** The protein level of ALOX15, FSP1, GDF15, GCH1 and GPX4 was tested by Western Blotting (*n* = 3). Values represent mean ± SD. Statistical analysis by one-way ANOVA followed by Tukey’s multiple comparisons test. *p < 0.05, **p < 0.01, ***p < 0.001.

Although reversing the down-regulation of GPX4 can effectively delay the course of ALS [16–18], the existence of ferroptosis in ALS still needs more verification. As an in vitro cell line for MNs in vitro, the NSC34 cells were chosen to validate the results of bioinformatics analysis. ALS model was successfully established by stably transfecting hSOD1^G93A^. For preliminary judgment on cell death, the CCK8 method was used to observe the response to reagents. After 2 h of incubation with 2 μM RSL3 (a ferroptosis inducer that inhibits GPX4), the ferroptosis in NSC34 cells could be specifically controlled by DFO and Fer-1 (classical ferroptosis inhibitor), but unchanged with Zvad (apoptosis inhibitor for caspase) and Nec-1 (necroptosis inhibitor for RIPK1) (Fig. 2A). H_2_O_2_ administration has been widely used as a substitute model for ALS to mimic exogenous oxidative stress in apoptosis and DNA damage studies [36, 37]. Conversely, Zvad and Nec-1 exhibited protective effects on cells with H_2_O_2_-induced apoptosis, whereas ferroptosis inhibitor Fer-1 had no effect (Fig. 2B). Nevertheless, inhibitors of both apoptosis and ferroptosis enhanced the viability of hSOD1^G93A^ cells (Fig. 2C). Furthermore, combining these two inhibitors showed notable higher viability than any other combinations or any individual reagent (Fig. 2C). These results indicate a contribution of both apoptosis and ferroptosis to ALS.

For systematical research, the generally accepted features including iron accumulation, lipid peroxidation, and GSH consumption involved in ferroptosis were tested [4]. Firstly, obvious lipid peroxidation was observed in hSOD1^G93A^ compared with WT as measured by the C11-BODIPY fluorescent probe, and it could be limited by ferroptosis inhibitors (Fig. 2D, E). As illustrated in photos of Electron microscopy, hSOD1^G93A^ resulted in the same mitochondrial reduction, increase of membrane density, and ridge destruction as ferroptosis induction (Fig. 2F). Besides, the elevated LIP was affirmed by the Calcein AM test (Fig. 2G). And with the detection kit, an abnormal decrease of GSH also appeared in hSOD1^G93A^ compared with control (Fig. 2H). Further, expression of the screened ferroptosis DEGs and critical genes in the current research was verified by qRT-PCR and Western blots. The mutant led to the up-regulated ALOX15 and GDF15 (respectively a driver and a marker of ferroptosis), down-regulated GCH1 and GPX4, and unaltered FSP1 (Figs. 1C and 2I–K). These results systematically prove ferroptosis involved in ALS and relevant features.

### MDM2 degrades SPY1 through ubiquitination in ALS

**Fig. 3:**
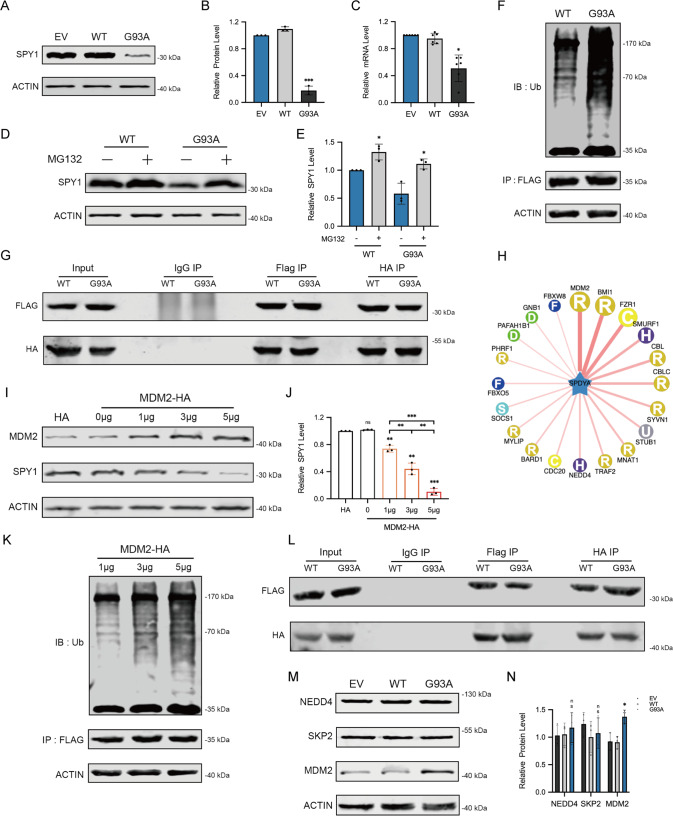
The decrease of SPY1 in ALS is mediated by MDM2. **A**, **B** Western blots and quantification for comparing the protein level of SPY1 in the cells of EV, WT, and hSOD1^G93A^ (*n* = 3). **C** The relative mRNA level of SPY1 was assessed by qRT-PCR (*n* = 6). **D**, **E** MG132 (25 μM) was added into hSOD1^G93A^ and WT cells for 6 h to evaluate the effect on expression of SPY1 (*n* = 3). **F** After transfection with SPY1-Flag for 24 h and incubation with MG132 (10 μM) for 4 h in cells of hSOD1^G93A^ and WT, the supernatants of cell lysates were immunoprecipitated with anti-Flag and immunoblotted with anti-ubiquitin. **G** After transfection with SPY1-Flag and SKP2-HA for 24 h, the supernatants of cell lysates were immunoprecipitated with Flag (left) or HA (right) antibodies and immunoblotted with HA (lower panel) or Flag (upper panel) antibodies. **H** UbiBrowser was used to predict E3 ligase interacted with SPY1 as substrate. The prediction score of interaction was expressed from high to low by connecting lines from thick to fine. **I**, **J** Western blots and quantification for changes of SPY1 in NSC34 cells transfected with different doses of MDM2-HA (*n* = 3). **K** Alteration in ubiquitination of SPY1 with the increase of transfected MDM2. IP, anti-Flag. IB, anti-ubiquitin. **L** After transfection with SPY1-Flag and MDM2-HA for 24 h, immunoprecipitated with Flag (left) or HA (right) antibodies and immunoblotted with HA (lower panel) or Flag (upper panel) antibodies. **M**, **N** Western blots and quantification for NEDD4, SKP2, and MDM2 in the cells of EV, WT, and hSOD1^G93A^ (*n* = 3). Values represent mean ± SD. Statistical analysis by one-way ANOVA followed by Tukey’s multiple comparisons test. *p < 0.05, **p < 0.01, ***p < 0.001.

To explore the reason for the decrease of SPY1 in ALS, transcriptional and translational levels were detected by qRT-PCR and Western blots showing that the decreased proportion of mRNA was not as obvious as protein (approximately 46.3% vs. 83.8%) (Fig. 3A–C). Proteins are considered as important functional executors. Ubiquitin-proteasome system and autophagy-lysosome pathway are the major mechanisms for protein degradation. In further test, the expression of SPY1 was raised by administration of MG132 (proteasome inhibitor) rather than pepstatin A with E64D (PE, autophagy-lysosome inhibitor) (Figs. 3D, E and S1A, B). Both E3 ligase SKP2 and NEDD4 have been reported to interact with SPY1 through ubiquitination [38, 39]. As expected, the ubiquitination level of SPY1 was significantly elevated in hSOD1^G93A^ (Figs. 3F and S1C). However, there was no significant increase of interaction between SPY1 and SKP2 or NEDD4 detected by co-immunoprecipitation (CoIP) (Figs. 3G and S1D–H). To find the true protein that mediated SPY1 ubiquitination in ALS, the sequence of amino acids was adopted to predict the E3 ligase with corresponding combined structure through the UbiBrowser. Among the results, MDM2 obtained the highest score of prediction (Fig. 3H). Strikingly, both SPY1 and its ubiquitination were increased in a dose-dependent manner with transfection of MDM2, and the degradation of SPY1 was inhibited in knockdown experiment. (Figs. 3I–K and S1I–K). In addition, knockdown of MDM2 enhanced the viability of hSOD1^G93A^ cells (Fig. S1L). More exciting was that CoIP confirmed the enhanced exogenous and endogenous interaction between SPY1 and MDM2 in hSOD1^G93A^ compared with WT (Figs. 3L and S1M–Q). The fact that MDM2 mediates ubiquitination of SPY1 was also verified in an in vitro assay (Fig. S2A). To locate specific sites among the 27 potential lysine ubiquitination sites, we examined the ubiquitination levels of the three structural domains of SPY1 separately but with no significant difference (Fig. S2B–E). Additionally, the expression of MDM2 in hSOD1^G93A^ was significantly increased along with the unchanged NEDD4 and SKP2 (Fig. 3M, N). These results clarify that the elevated MDM2 mediates ubiquitination of SPY1, which mainly leads to decreased expression in ALS.

### Overexpression of SPY1 inhibits ferroptosis in ALS

**Fig. 4:**
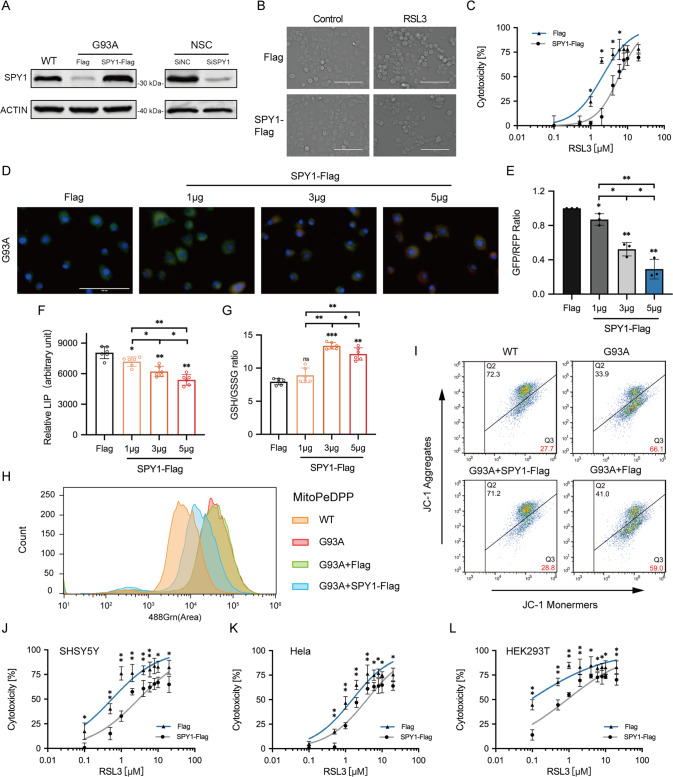
Overexpressed SPY1 resists ferroptosis in the cell model of ALS. **A** The effect of SPY1-Flag or SiSPY1 transfection in hSOD1^G93A^ or NSC34 cells was evaluated by Western blotting. **B** Microscopic images were used to show response to RSL3 (2 μM for 2 h) of NSC34 cells with SPY1-Flag and Flag. Scale bar, 100 μM. **C** The effect of overexpressed SPY1 on ferroptosis induction was assessed by LDH assay in NSC34 cells with the treatment of diverse dosages of RSL3 for 2 h (*n* = 6). **D**, **E** Images and quantification of C11-BODIPY fluorescence exhibited the level of lipid oxidation with the increase of transfected SPY1-Flag in hSOD1^G93A^ cells (*n* = 3). Scale bar, 100 μM. **F**, **G** The effect of increased SPY1 on relative LIP and GSH was shown (*n* = 6). **H** The lipid peroxidation of inner mitochondria membrane was measured by MitoPeDPP immunofluorescence staining followed by flow cytometry (channel 488, *n* = 3). **I** The changes in mitochondrial membrane potential caused by mutant SOD1 and overexpressed SPY1 were detected by JC-1 fluorescence staining followed by flow cytometry. **J**–**L** The effect of overexpressed SPY1 on RSL3-induced ferroptosis in SHSY5Y, Hela, and HEK293T cells was measured by LDH assay (*n* = 6). Values represent mean ± SD. Statistical analysis by one-way ANOVA followed by Tukey’s multiple comparisons test. *p < 0.05, **p < 0.01, ***p < 0.001.

To identify the relationship between SPY1 and ferroptosis, the overexpression and knockdown of SPY1 were constructed respectively in hSOD1^G93A^ and NSC34 cells (Fig. 4A). Previous experiments have shown the rescue of SPY1 to the apoptosis of hSOD1^G93A^ cells [27]. The decrease of viability caused by knockdown of SPY1 showed a comparative improvement by ferroptosis and apoptosis inhibitors (Fig. S3A, B). Knockdown of SPY1 also led to lipid peroxidation and overloaded labile iron (Fig. S3C–E). By contrast, overexpression was verified to enhance the resistance to RSL3-induced ferroptosis in NSC34 cells (EC50 = 5.873 vs. 2.058 µM) and also reduce the death of hSOD1^G93A^ cells(Figs. 4B, C and S3F). And the reduction of lipid peroxidation and free iron were dose-dependent on the transfection of SPY1 in hSOD1^G93A^ cells (Fig. 4D–F). In spite of increased GSH, it did not correlate with the transfection dosage (Fig. 4G). The protection to mitochondrial function by overexpression of SPY1 was performed via reducing the fluorescence of mitochondrial lipid peroxidation and alleviating the decline of mitochondrial membrane potential (MMP) (Figs. 4H, I and S3G). These results conclude that SPY1 is an effective inhibitor of ferroptosis in ALS. Additionally, its’ resistance to RSL3-induced and Erastin (another ferroptosis inducer that inhibits xCT for reverse transport of cystine and glutamate)-induced ferroptosis also occurs in other tumor cells (Figs. 4J–L and S3H, I). However, SHSY5Y cells and NSC34-derived WT and hSOD1^G93A^ cells were not sensitive to Erastin (Fig. S3J, K).

### SPY1 resists ferroptosis by regulating GCH1/BH4 and TFR1

**Fig. 5:**
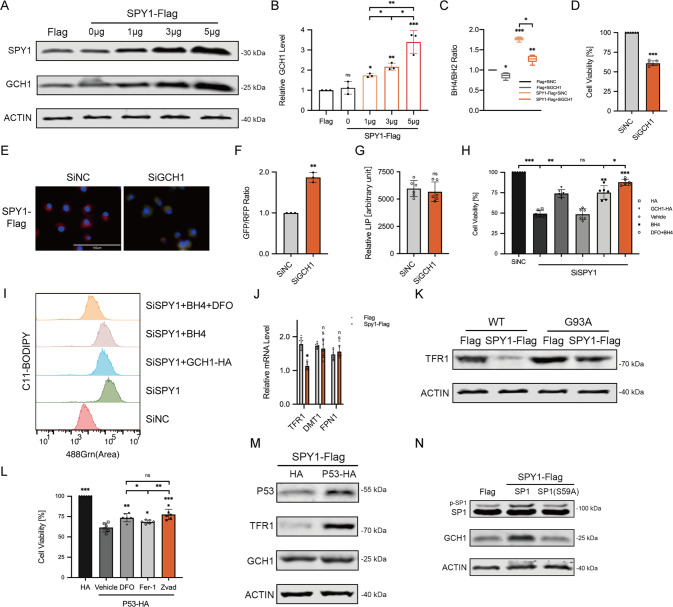
SPY1 inhibits ferroptosis through regulatory of GCH1/BH4 and TFR1. **A**, **B** Western blots and quantification for changes of GCH1 in hSOD1^G93A^ cells transfected with different doses of SPY1-Flag (*n* = 3). **C** The effect of transfected SPY1-Flag and SiGCH1 on relative content of BH4 was shown (*n* = 6). **D**–**G** The effect of knockdown GCH1 on viability, lipid oxidation, and LIP in overexpressed SPY1 cells compared with control (in **D** and **G**, *n* = 6; in **F**, *n* = 3). Scale bar, 100 μM. **H** The viability was measured in SiSPY1 cells with transfected GCH1-Flag and treatment of BH4 (50 μM) and DFO (100 μM) for 24 h (*n* = 6). **I** The extent of lipid peroxidation was assessed by C11-BODIPY fluorescence staining followed by flow cytometry (channel 488). **J** The relative mRNA of TFR1, DMT1 and FPN1 in SPY1-Flag cells compared with control (*n* = 6). **K** Western blots for TFR1 in WT and hSOD1^G93A^ cells with overexpressed SPY1 or control were shown. **L** After sequential transfection with P53-HA and control and incubation with DFO (100 μM), Fer-1 (10 μM), Zvad (20 μM), and vehicle for 24 h. The viability of SPY1-Flag cells was assessed by CCK8 assay (*n* = 6). **M** Western blots for P53, TFR1, and GCH1 in SPY1-Flag cells with overexpressed P53 and control were shown. **N** Western blots were shown for expression of p-SP1, SP1, and GCH1 in SPY1-Flag cells with overexpressed SP1(S59A) and wild type. Values represent mean ± SD. Statistical analysis by one-way ANOVA followed by Tukey’s multiple comparisons test. *p < 0.05, **p < 0.01, ***p < 0.001.

In order to further explore the mechanism of SPY1 regulating ferroptosis, the mRNA of vital genes altered in ALS was firstly screened in cells with overexpressed SPY1 and control. With the addition of the expression correlation analysis and functional annotation in SPY1 High vs. Low, the up-regulated ferroptosis suppressor GCH1 was speculated to be responsible for resistance to ferroptosis (Figs. 1D–G and S4A). Subsequently, the translational level of GCH1 was confirmed up-regulated in a dose-dependent manner with overexpressed SPY1 transfection (Fig. 5A, B). Plasmids for overexpression and knockdown of GCH1 were established (Fig. S4B). Besides, overexpression of GCH1 was unable to affect SPY1 inversely (Fig. S4C). GCH1 is a rate-limiting enzyme for the synthesis of BH4 which was significantly elevated by overexpression of SPY1 as well and turned decreased after the knockdown of GCH1 (Fig. 5C). Similarly, changes in viability and lipid oxidation induced by overexpressed SPY1 were reversed with knockdown of GCH1 (Fig. 5D–F). However, the LIP was found irrelevant to expression of GCH1 (Fig. 5G). Although administration of BH4 and overexpression of GCH1 protected most viability and lipid peroxidation in SPY1-knockdown cells (Fig. 5H, I). It is known that free iron plays an important role in generating lipid peroxidation, the combination of DFO and BH4 was casually tested and unexpectedly displayed an additional amelioration(Fig. 5H, I).

So as to seek the modulation of labile iron independent on GCH1/BH4 axis, the transcriptional level of iron transport-related genes was detected in cells of overexpressing SPY1. Compared with control, the mRNA of TFR1 responsible for transporting iron into cells was increased along with unchanged DMT1 and FPN1 which were respectively accounting for intracellular iron to release and export (Fig. 5J). Consistent with mRNA, the protein level exhibited that overexpression of SPY1 lowered the abnormally elevated TFR1 in hSOD1^G93A^ (Figs. 5K and S4D). Moreover, the LIP restored by TFR1 led to lipid peroxidation and cell death of overexpressed SPY1 cells (Fig. S4E–H).

As a multi-functional transcription factor, P53 has been demonstrated to be downregulated by SPY1 in our previous study [27]. To determine whether SPY1’s resistance to ferroptosis is mediated by regulating P53, we examined the effects of overexpressing P53 in SPY1-Flag cells on viability and related proteins. It was found that the inverse cell death caused by overexpressed P53 could be rescued by apoptosis inhibitors as well as by ferroptosis inhibitors, especially DFO implying a link between P53 and the transport of free iron (Fig. 5L). And the expression of TFR1 rather than GCH1 was upregulated with the overexpression of p53 (Figs. 5M and S4I). Studies have proposed that SP1 is likely to be a transcriptional activator of GCH1, and the phosphorylation of SP1 mediated by CDK2 can activate downstream transcriptional activity [40]. After expressing the mutation of SP1 (S59A), we found that SPY1 could activate GCH1 by upregulating the phosphorylation level of SP1 (Figs. 5N and S4J). Taken together, the restraint of SPY1 on ferroptosis attributes to inhibiting lipid peroxidation by regulating GCH1/BH4 axis and TFR1 expression.

### SPY1 protects mouse model of ALS by inhibiting neuronal ferroptosis

**Fig. 6:**
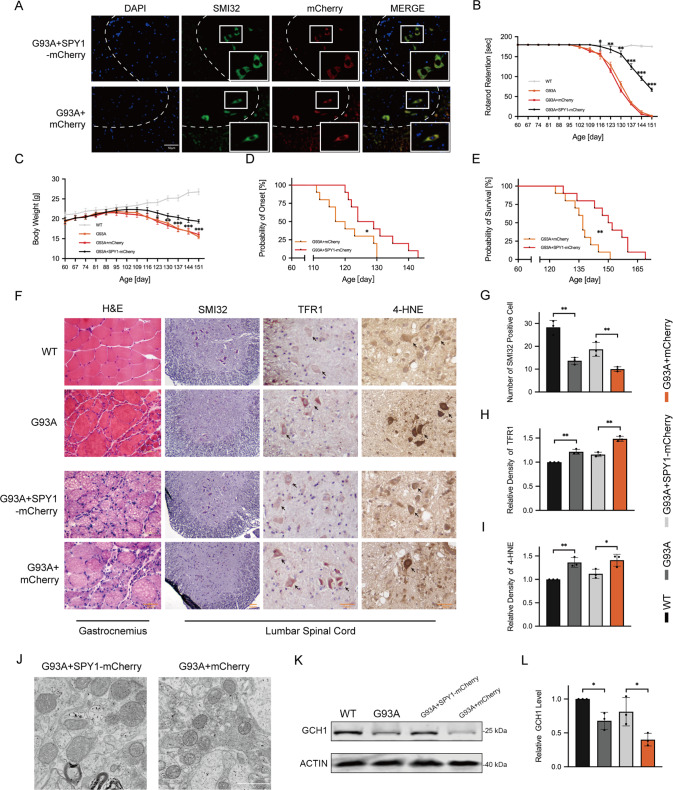
SPY1 plays a protective role in hSOD1G93A mice by resisting neuronal ferroptosis. hSOD1^G93A^ transgenic mice at 60 d were injected with AAV9-hSyn-SPY1-mCherry and AAV9-hSyn-mCherry. **A** The overexpressed SPY1 in green MNs of lumbar spinal cord stained with SMI32 was verified by autofluorescence of mCherry at 90 d. Scale bar, 50 μM. **B**, **C** The decreased body weight and rotarod test were recorded (*n* = 10). **D**, **E** Kaplan–Meier survival analysis for probability of onset and survival (*n* = 10). **F** Images of immunohistochemical staining in end-stage mice. First column for the gastrocnemius muscle stained with H&E. The residue for the lumbar spinal cord tissue immunohistochemically stained with SMI32, TFR1, and 4-HNE. Scale bar, 50 μM. **G**–**I** Quantification for the number of SMI32 positive cells and relative density of TFR1 and 4-HNE (*n* = 3). **J** Electron microscopy for mitochondrial changes of the lumbar spinal cord in hSOD1^G93A^ mice with overexpression of SPY1 and control. Scale bar, 1 μM. **K**, **L** Western blots and quantification for the effect of mutant SOD1 and overexpressed SPY1 on the expression of GCH1 in lumbar spinal cord (*n* = 3). Values represent mean ± SD. Statistical analysis by one-way or two-way ANOVA followed by Tukey’s post hoc tests. *p < 0.05, **p < 0.01, ***p < 0.001.

To demonstrate the impact of SPY1-overexpression on the progression of ALS in vivo, the recombinant virus targeting neuron-specific expression was injected into the lateral ventricle of hSOD1^G93A^ mice (Fig. 6A). Progressive muscle weakness and atrophy are the main manifestations of ALS. To evaluate the status of muscles, rotarod test and body weight were monitored during the course. The decline of exercise capacity was postponed in hSOD1^G93A^ mice with SPY1-overexpression (Fig. 6B). And the occurrence of weight loss was also delayed for approximately 2 weeks (Fig. 6C). The degree of muscle weakness and muscle atrophy are dominant factors affecting the onset and survival of ALS. Compared with control, the overexpression of SPY1 retarded the onset (128.90 ± 8.17 vs. 120.40 ± 7.28 d) and prolonged the survival (149.90 ± 12.72 vs. 136.80 ± 7.98 d) (Fig. 6D, E).

Next, tissues of gastrocnemius and lumbar spinal cord from mice at 150 d were performed histochemical staining to elucidate the protective mechanism of SPY1. It was shown that SPY1 significantly delayed the neurogenic atrophy in hSOD1^G93A^ mice corresponding to the rotarod test (Fig. 6F). As a specific marker of MNs, SMI32 staining in the anterior horn of the spinal cord revealed that SPY1 reduced the loss of MNs (Fig. 6F, G). The increased expression of TFR1 itself is strong evidence of iron accumulation which was found in hSOD1^G93A^ and corrected by SPY1 (Fig. 6F, H) [7]. Additionally, immunohistochemical results of 4-HNE, a marker of lipid oxidation, showed that the initially elevated level of lipid peroxidation was decreased in mice overexpressing SPY1 (Fig. 6F, I). Moreover, SPY1 maintained healthier mitochondria with relative normal morphology (Fig. 6J). Having verified the expression of TFR1 by immunohistochemistry, GCH1 was proved down-regulated in hSOD1^G93A^ mice and its activation by overexpressed SPY1 (Fig. 6K, L). These results obtained from hSOD1^G93A^ mice are consistent with those in vitro, emphasizing that SPY1 prolongs survival by inhibiting neuronal ferroptosis in ALS.

## Discussion

The death of motor neurons in ALS has always been controversial. Previous studies on the hSOD1^G93A^ model mainly focused on apoptosis. Whereas embryonic stem-cell-derived MNs co-cultured with sporadic ALS (sALS) astrocytes were found to be triggered to degenerate through necroptosis rather than apoptosis dependent on caspases [41]. This distinction may be related to the uncertain mechanism of sALS astrocytes used, because the silencing of SOD1 or TDP-43 exhibited no effects on the loss of MNs, which indicates quite different from traditional models. Moreover, there seems to be more controversy about necroptosis in ALS. Another study holding the same view detected the expression of RIPK1, RIPK3, and MLKL (main components of necroptosis) in ALS patients and mouse models with mutant OPTN and SOD1, and concluded that the limitation of RIPK3 or RIPK1 protected ALS [42]. While diverse results were shown in the other three studies [43–45]. In particular, RIPK3 and MLKL have recently been proved undetected in central nervous system tissues of healthy or hSOD1^G93A^ mice [16]. Consistent with our study, the RIPK1 inhibitor Nec-1 displayed no effects on hSOD1^G93A^ cells, so we temporarily do not support the existence of necroptosis especially in hSOD1^G93A^ model. In terms of ferroptosis in our research, its correlation with ALS was firstly concluded by the enrichment of bioinformatics analysis. Same as Dixon’s results in HT-1080 cells [4], Fer-1 exhibited no effect on NSC34 cells with H_2_O_2_ administered which is usually used to study apoptosis. Similarly, apoptosis inhibitor has no impact on induced ferroptosis. Both inhibitors showed a comparable specificity and obvious protection in the model of hSOD1^G93A^. Meanwhile, compared with hSOD1^G93A^, it is suggested that the oxidative stress model H_2_O_2_ still cannot fully replace the mutation. Furthermore, the lipid peroxidation in hSOD1^G93A^ could be alleviated by ferroptosis inhibitors. Hence we reasonably infer that ferroptosis exists in ALS.

As for current results, lipid peroxidation involved in mutant hSOD1^G93A^-induced ferroptosis attributes to excess free iron imported by TFR1, decreased GSH, mitochondrial membrane dysfunction, upregulated ALOX15, and inactivation of GCH1, GPX4. The mRNA of DMT1 and TFR1 has been shown upregulated in SH-SY5Y cells with hSOD1^G93A^ [7]. And similar results were also found in the cervical spinal cord of SOD1G37R mice at end-stage [46]. Despite focusing on the role of SPY1, the only detected expression of TFR1 is still enhanced in accordance with previous studies. Besides being involved in the Fenton reaction, the catalyzer iron bound with upregulated lipoxygenase ALOX15, which is a marker and also a driver of ferroptosis, promotes the stereo-specific peroxidation of free and esterified polyunsaturated fatty acids generating a spectrum of bioactive lipid mediators [47]. Accompanied by the decline of GPX4 consistent with preceding research [16–18], the new resistance factor GCH1 was verified downregulated demonstrating a unique dysfunctional mechanism of protecting phosphatidylcholine phospholipids through two polyunsaturated fatty acyl chains and increasing the level of coenzyme Q10 that is independent of the GSH/GPX4 system [48]. However, we have also detected the upregulation of ferroptosis suppressor GDF15 which seems not ought to emerge. The suppressive role was derived from a study in which its knockout can promote Erastin-induced ferroptosis by decreasing xCT expression [49]. But it has been shown in ALS that xCT expression is enriched in microglia compared to total mouse spinal cord and absent from motor neurons [50]. So the upregulated GDF15 by the mutation of hSOD^G93A^ can not rescue the various features of ferroptosis, even including functional defects in xCT/GSH system and still needs functional study in ALS. In addition, it is noted that Erastin can not result in death of hSOD1^G93A^ or WT cells. Indeed, it is consistent with a previous study in which the resistance to Erastin in NSC34 and SHSY5Y cells is considered to be associated with the intact function of the xCT system [51]. Considering that Erastin still fails to induce ferroptosis in hSOD1G93A cells with functional defects in xCT/GSH system, presumably there is some unknown resistance property to Erastin in NSC34 cells. But this does not affect the results of those altered ferroptosis characteristics caused by the mutation of hSOD1^G93A^ and the inhibition by SPY1.

It has been reported that the proteasome degrades SPY1 in U2OS cells [21]. Having excluded the autophagy-lysosome pathway, our experiments demonstrate the ubiquitin-proteasome system for SPY1 to degrade in MNs. CoIP has assisted us to rule out the possibility that the increased ubiquitination is mediated by NEDD4 and SKP2. This is probably because of no changes in the expression of both in ALS compared with WT. Although different domains of SPY1 were used to detect ubiquitination levels in order to narrow the search for specific ubiquitination sites, the insignificant results may suggest the presence of polyubiquitination sites in SPY1 and still need further study.

Via data analysis and validation in vitro and in vivo experiments, our study is the first to find the relationship between SPY1 and ferroptosis. SPY1 was regarded as an apoptosis suppressor by inhibiting p53-mediated DNA damage in previous studies [27, 52]. In the part of bioinformatics analysis, the DEGs of SPY1 High vs. Low were significantly enriched in negative regulation of apoptosis, which also suggests relatively reliability of data. In vitro, a similar protective effect was found in SPY1 knockdown cells with specific apoptosis and ferroptosis inhibitors. Especially the effective Fer-1 has no impact on H_2_O_2_-induced apoptosis and DNA damage, implying additional ferroptosis involved in SPY1 knockdown. Therefore, we do not exclude that SPY1 has other protective effects on ALS while resisting ferroptosis. In accordance with the reasonable screening of transcriptome data and verification in experiments, we confirm SPY1 as a new upstream activator of GCH1/BH4 pathway to control most of the lipid peroxidation in ALS. However, the reduced level of free iron by SPY1 was not mediated through GCH1. In addition, the combination of DFO and BH4 had a stronger effect on cell viability and lipid peroxidation, suggesting that the decrease of partial lipid peroxidation produced by free iron is also an important mechanism for SPY1 to resist ferroptosis. Among the genes related to iron transport, the downregulated TFR1 was detected to lessen excess labile iron. Considering the resistance of SPY1 to GPX4 inhibitor RSL3 in NSC34 and different tumor cells, we believe that SPY1 and downstream pathways may be effective protectors against ferroptosis caused by GPX4 deletion. Besides, there may be no direct regulatory relationship between SPY1 and the level of GSH, but improved lipid peroxidation reduces the consumption of partial GSH to resist ferroptosis induced by Erastin in other tumors.

SPY1/CDK2 complex, the unique binding motivates CDK2 phosphokinase activity, which is likely to affect the status of transcription factors through phosphorylation [23]. Chromatin immunoprecipitation assays have revealed that proteins that bind in vivo to the endogenous GCH1 proximal promoter actively transcribing GCH1 within living PC12 cells, including SP1 [53, 54]. The phosphorylation of SP1 mediated by CDK2 has been reported to activate downstream CTalpha transcription [40]. Moreover, our previous experiments have confirmed the interaction between SPY1 and CDK2 in hSOD1^G93A^ cells [27]. In our study, functional enrichment of the gene SPY1 in ALS showed a positive regulation of transcription which also lays a foundation for SPY1 to regulate the expression of GCH1 by phosphorylating SP1. In addition, inhibition or knockdown of USP7 enhanced the ubiquitination of p53 along with the decreased levels of p53, which led to down-regulation of TFR1 [55]. Our results also demonstrate that SPY1 regulates TFR1 through the inhibition of P53.

## Conclusion

In conclusion, our results demonstrate that ferroptosis is involved in the pathogenesis of ALS. The decreased SPY1 in ALS is due to the ubiquitination mediated by interaction with MDM2. Furthermore, SPY1 inhibits ferroptosis by regulating GCH1/BH4 axis and TFR1 to delay the occurrence and progression of ALS.

## Supplementary information

Supplementary Figure 1

Supplementary Figure 2

Supplementary Figure 3

Supplementary Figure 4

Western blot original image

Author contribution

Reproducibility checklist

Supplementary Figure Legends

## Data Availability

All data are available from the corresponding author upon reasonable request. Supplementary figures and original Western blots images are available at Supplementary Materials.
