# Supplementary figures and images for: SPY1 inhibits neuronal ferroptosis in amyotrophic lateral sclerosis by reducing lipid peroxidation through regulation of GCH1 and TFR1
Source: Cell Death Differ. 2022 Nov 28;30(2):369–82. doi: 10.1038/s41418-022-01089-7 (PMC9950139; doi:10.1038/s41418-022-01089-7)

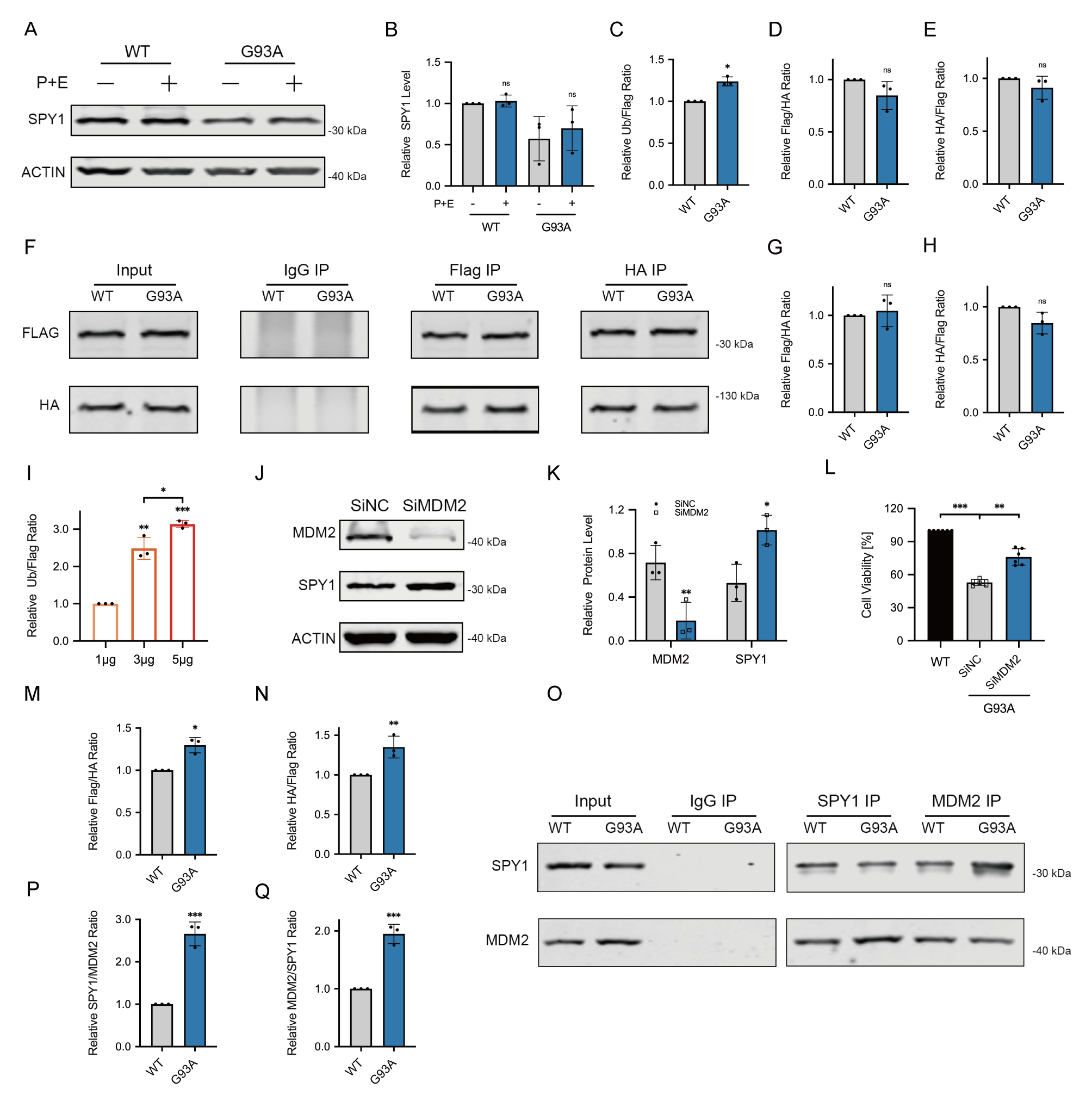

Supplement: Supplementary file 1 — Supplementary Figure 1 [file 41418_2022_1089_MOESM1_ESM.png]

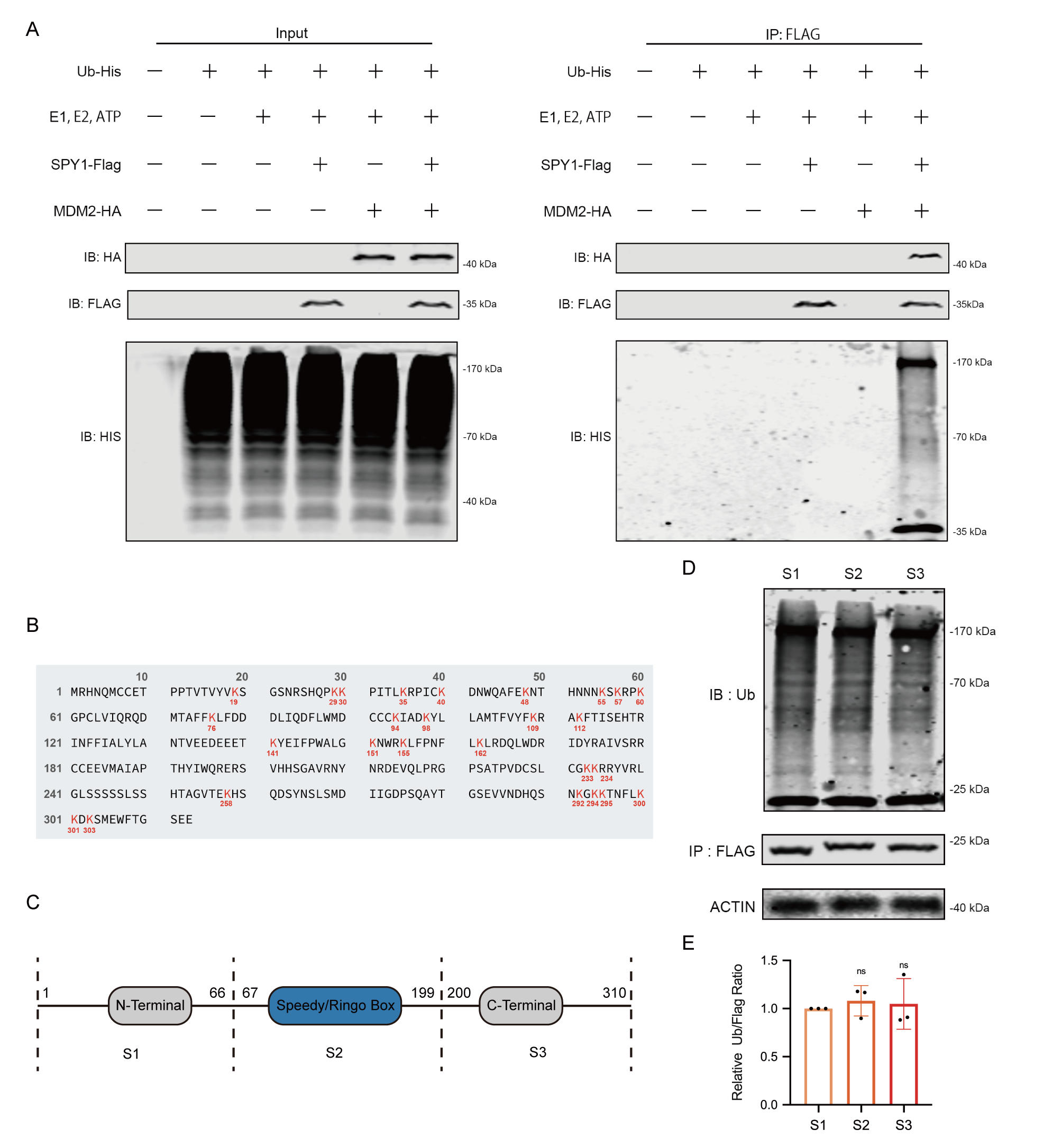

Supplement: Supplementary file 2 — Supplementary Figure 2 [file 41418_2022_1089_MOESM2_ESM.png]

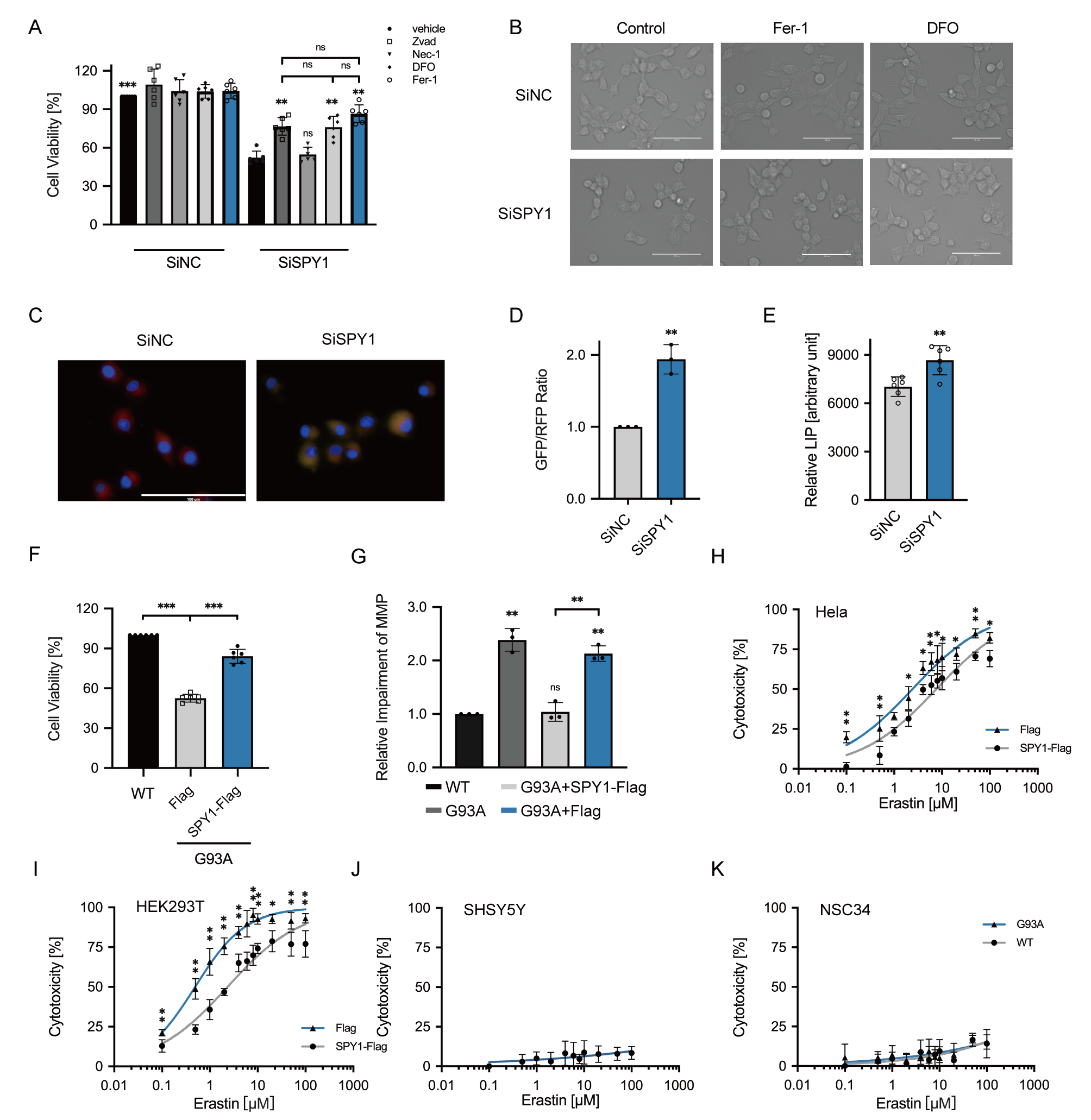

Supplement: Supplementary file 3 — Supplementary Figure 3 [file 41418_2022_1089_MOESM3_ESM.png]

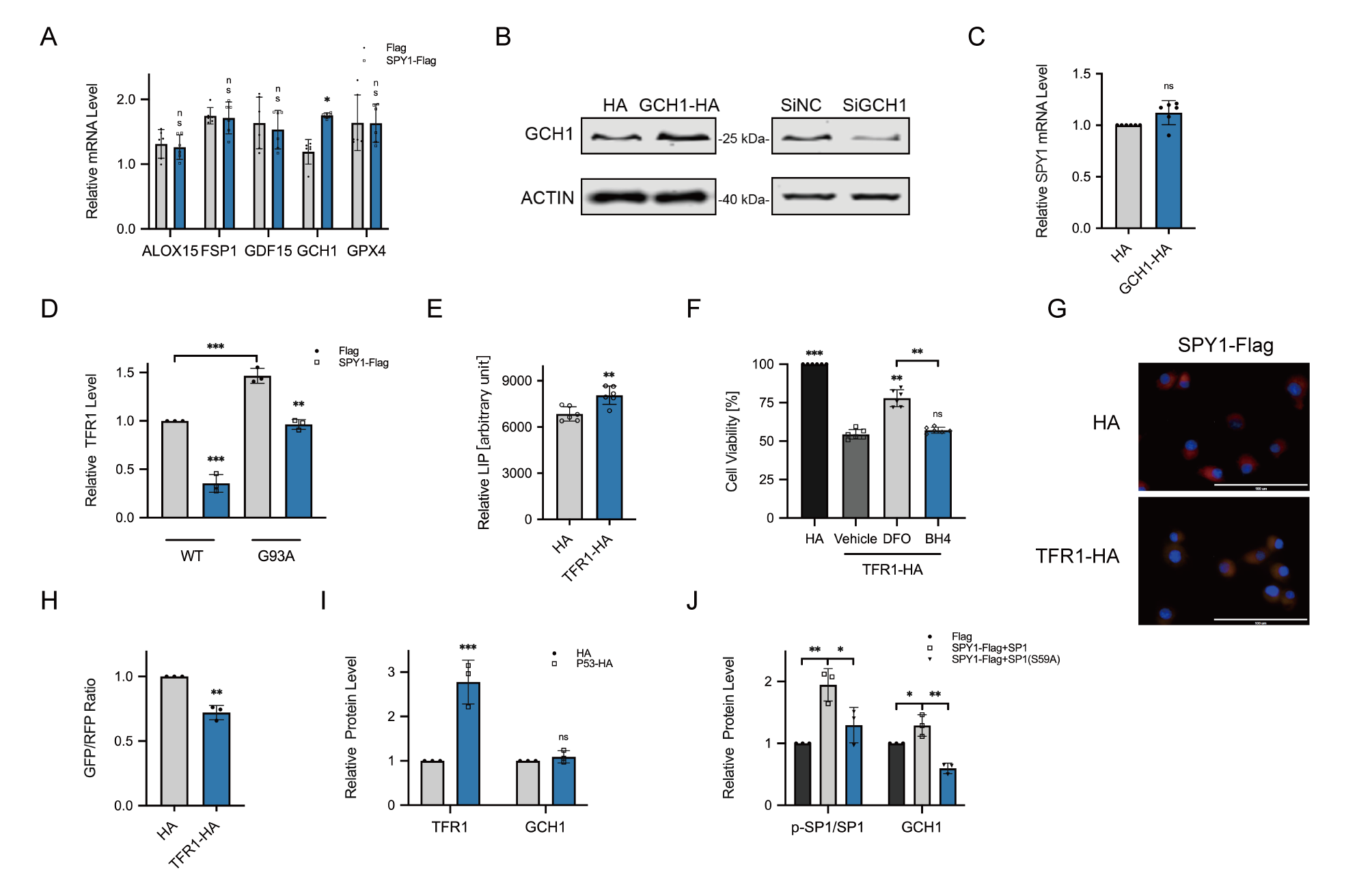

Supplement: Supplementary file 4 — Supplementary Figure 4 [file 41418_2022_1089_MOESM4_ESM.png]
