# Supplementary material for: SPY1 inhibits neuronal ferroptosis in amyotrophic lateral sclerosis by reducing lipid peroxidation through regulation of GCH1 and TFR1
Source: Cell Death Differ. 2022 Nov 28;30(2):369–82. doi: 10.1038/s41418-022-01089-7 (PMC9950139; doi:10.1038/s41418-022-01089-7)
Supplement: Supplementary file 6 — Author contribution [file 41418_2022_1089_MOESM6_ESM.pdf]

DECLARATION OF CONTRIBUTIONS TO ARTICLE

ADMC

Manuscript Number: CDD-022-0659

Journal Name: *Cell Death & Differentiation* (the 'Journal')

Proposed Title of the Contribution: Contribution to the article (the 'Contribution)

Author(s): Wang D, Liang W, Huo D, et al. (the 'Authors')

For all *CDD* articles, each person named as an author in the published version must be able to show he or she has contributed substantially to the article.

Authorship credit should be based on 1) substantial contributions to conception and design, acquisition of data, or analysis and interpretation of data; 2) drafting the article or revising it critically for important intellectual content; and 3) final approval of the version to be published. Authors should meet conditions 1, 2 and 3.

Any person who cannot be shown to have made a substantial contribution to the article cannot be listed as an author in the final version. The name of any person who is deemed to have made a minor contribution can, however, appear in the Acknowledgments section of the article.

Please complete the table below to indicate the contributions of all named authors to the manuscript.

Author Full Name:	Specification of Contribution to the Manuscript:
Di Wang	idea, main experiments and manuscript writing
Weiwei Liang	cell experiment and immunofluorescence
Di Huo	animal experiment and manuscript revise
Hongyong Wang	tissue extraction and staining
Ying Wang	cell experiment and qRT-PCR
Chaohua Cong	animal experiment and qRT-PCR
Chunting Zhang	idea, cell and animal experiment
Shi Yan	bioinformatics analysis and pictures drawing
Ming Gao	cell experiment and western blot
Xiaoli Su	cell experiment and manuscript revise
Xingli Tan	animal experiment and figure arrangement
Wenmo Zhang	data collation and statistics
Honglin Feng	idea, manuscript writing and revise

Please complete the table below to indicate the contributions of all named authors to the figures.

Figure 1:

Di Wang got the data and provided the idea for analysis.
Shi yan used R to process the data and drawed pictures.
Ling Han and Dongmei Zhang arranged figure.
Di wang modified and improved figure.

Figure 2:

Honglin Feng provided idea and guided direction.
Di Wang conducted CCK8, GSH, and LIP assay.
Xingli Tan arranged figure.
Weiwei Liang cultured cells and conducted immunofluorence assay.
Ming Gao conducted mitochondrial electron microscope assay.
Xiaoli Su conducted western blot.

Figure 3:

Di Wang conducted ubiquination assay and CoIP assay.
Wenmo Zhang carried out statistical analysis.
Weiwei Liang helped complete western blot.
Shi Yan predicted the interaction by bioinformatics.
Chunting Zhang cultured cells and conducted drug incubation assay.

Figure 4:

Ying Wang conducted LDH assay and helped complete western blot.
Chunting Zhang conducted transfection assay and flow cytometry assay.
Di Wang and Ming Gao conducted lipid oxidation, LIP, and GSH assay.
Di Huo cultured cells and arranged figure.

Figure 5:

Di Wang and Weiwei Liang conducted transfection and western blot assay.
Honlin Feng provided direction of study.
Chaohua Cong conducted the qRT-PCR assay.
Ling Han drawed pictures and arrange the figure.
Wenmo Zhang cultured cells and analysed the statistics.

Figure 6:

Hongyong Wang fed animals, extracted tissues from mice and stainned the tissue.
Di Huo identified positive mice by PCR and collected behavior data.
Ying wang injected virus to build mice model.
Di Wang and Chaohua Cong conducted western blot.
Ming Gao conducted tissue electron microscope assay.

Signed for and on behalf of the Author(s):	Print Name:	Date:
Honglin Feng	Honglin Feng	September 19, 2022
