# Supplementary material for: SPY1 inhibits neuronal ferroptosis in amyotrophic lateral sclerosis by reducing lipid peroxidation through regulation of GCH1 and TFR1
Source: Cell Death Differ. 2022 Nov 28;30(2):369–82. doi: 10.1038/s41418-022-01089-7 (PMC9950139; doi:10.1038/s41418-022-01089-7)
Supplement: Supplementary file 8 — Supplementary Figure Legends [file 41418_2022_1089_MOESM8_ESM.docx]

**Supplementary Figure 1.**

(A and B) Pepstatin A (10 μg/ml) and E64D (10 μg/ml) were added to hSOD1^G93A^ and WT cells for 6 h to evaluate the effect on the expression of SPY1 (n = 3). (C) Quantification for immunoblotting with anti-ubiquitin of the cell lysates immunoprecipitated with anti-Flag (n = 3). (D and E) Quantification for immunoblotting with HA or Flag antibodies of cell lysates immunoprecipitated with Flag or HA antibodies after transfection with SPY1-Flag and SKP2-HA for 24 h (n = 3). (F, G, and H) After transfection with SPY1-Flag and NEDD4-HA for 24 h, the supernatants of cell lysates were immunoprecipitated with Flag (left) or HA (right) antibodies and immunoblotted with HA (lower panel) or Flag (upper panel) antibodies (n = 3). (I) Quantification for alteration in ubiquitination of SPY1 with the increase of transfected MDM2 (n = 3). (J and K) Western blots and quantification for changes of SPY1 in hSOD1^G93A^ cells transfected with SiMDM2 and control (n = 3). (L) The effect MDM2-knockdown on cell viability was assessed by CCK8 assay after transfected with SiMDM2 in hSOD1^G93A^ cells for 48 h (n = 6). (M and N) Quantification for immunoblotting with HA or Flag antibodies of cell lysates immunoprecipitated with Flag or HA antibodies after transfection with SPY1-Flag and MDM2-HA for 24 h (n = 3). (O, P, and Q) The supernatants of cell lysates were immunoprecipitated with SPY1 (left) or MDM2 (right) antibodies and immunoblotted with MDM2 (lower panel) or SPY1 (upper panel) antibodies (n = 3). Values represent mean ± SD. Statistical analysis by one-way ANOVA followed by Tukey’s multiple comparisons test. **p <* 0.05, ***p <* 0.01, ****p <* 0.001.

**Supplementary Figure 2.**

(A) The purified proteins of SPY1-Flag (1 μg), MDM2-HA (1 μg), and Ub-His (2 μg) were reacted with E1, E2, and ATP to detect ubiquitination of SPY1. (B) All potential lysine ubiquitination sites of SPY1 were shown. (C) Three SPY1 structural domains with Flag tag were established. (D and E) After transfection with 3 Flag-labeled domains of SPY1 for 24 h and incubation with MG132 (10 μM) for 4 h in cells of hSOD1^G93A^, the supernatants of cell lysates were immunoprecipitated with Flag antibodies and immunoblotted with Ubiquitin antibodies (n = 3). Values represent mean ± SD. Statistical analysis by one-way ANOVA followed by Tukey’s multiple comparisons test. *p < 0.05.

**Supplementary Figure 3.**

1. Altered cell viability of hSOD1^G93A^ cells due to overexpression of SPY1 was shown using CCK8 assay. (B and C) Different Inhibitors’ effect on NSC34 cells with SiSPY1 and SiNC was detected by CCK8 assay and microscopy (n = 6). Scale bar, 100 μM. (D) The hSOD1^G93A^ and WT cells were treated with a diverse dosage of Erastin for 2 h to contrast sensitivity to ferroptosis induction by measurement of cell viability using CCK8 assay (n = 6). (E, F, and G) The effect of knockdown SPY1 on the lipid oxidation and LIP in NSC34 cells compared with control (in D, n = 3; in E, n = 6). Scale bar, 100 μM. (H) Quantification for changes of MMP (n = 3). (I) Effect of incubation with Erastin on the cytotoxicity of SHSY5Y cells using LDH assay (n = 6). (J and K) The effect of overexpressed SPY1 on Erastin-induced ferroptosis in Hela and HEK293T cells was measured by LDH assay (n = 6). Values represent mean ± SD. Statistical analysis by one-way ANOVA followed by Tukey’s multiple comparisons test. **p <* 0.05, ***p <* 0.01, ****p <* 0.001.

**Supplementary Figure 4.**

(A) The relative mRNA of ALOX15, FSP1, GDF15, GCH1, and GPX4 was evaluated in SPY1-Flag cells compared with control (n = 6). (B) The effect of GCH1-HA or SiGCH1 transfection in hSOD1^G93A^ or NSC34 cells was evaluated by Western blotting. (C) The relative mRNA of SPY1 was evaluated in GCH1-HA cells compared with control (n = 6). (D) Quantification for the effect of overexpressed SPY1 on the expression of TFR1 in hSOD1^G93A^ and control cells by Western blotting (n = 3). (E) The effect of increased TFR1 on relative LIP in SPY1-Flag cells (n = 6). (F) The effect of increased TFR1 with vehicle, DFO (100 μM), and BH4 (50 μM) on viability in SPY1-Flag cells (n = 6). (G and H) The extent of lipid oxidation in SPY1-Flag cells with TFR1-HA and control detected by C11-BODIPY immunofluorescence (n = 3). (I) Quantification for the effect of overexpressed P53 on the expression of TFR1 and GCH1 in SPY1-Flag cells by Western blotting (n = 3). (J) Quantification for the effect of SP1 phosphorylation level on the expression of GCH1 in SPY1-Flag cells by Western blotting (n = 3). Values represent mean ± SD. Statistical analysis by one-way ANOVA followed by Tukey’s multiple comparisons test. **p <* 0.05, ***p <* 0.01, ****p <* 0.001.
